# Retraction: Clinicopathologic and prognostic significance of tumor-associated macrophages in patients with hepatocellular carcinoma: A meta-analysis

**DOI:** 10.1371/journal.pone.0282201

**Published:** 2023-02-17

**Authors:** The PLOS ONE Editors

After this article was published, similarities were noted between this article and submissions by other research groups which call into question the validity and provenance of the reported results.

In light of these issues, the *PLOS ONE* Editors retract this article [1].

XX and WD did not agree with retraction. YT, YQ, WX, YW, and PJ either did not respond directly or could not be reached.
